# Proposed A_2_C_2_S_2_-VASc score for predicting atrial fibrillation development in patients with atrial flutter

**DOI:** 10.1136/openhrt-2020-001478

**Published:** 2021-01-29

**Authors:** Yung-Lung Chen, Hui-Ting Wang, Huang-Chung Chen, Wen-Hao Liu, Shaur-Zheng Chong, Shu-Kai Hsueh, Chang-Ming Chung, Yu-Shen Lin

**Affiliations:** 1 Division of Cardiology and Department of Internal Medicine, Kaohsiung Chang Gung Memorial Hospital, Kaohsiung, Taiwan; 2 Graduate Institute of Clinical Medical Sciences, College of Medicine, Chang Gung University, Taiwan, College of Medicine, Chang Gung University, Taoyuan, Taiwan; 3 Emergency Department, Kaohsiung Chang Gung Memorial Hospital, Kaohsiung, Taiwan; 4 Division of Cardiology, Chiayi Chang Gung Memorial Hospital, Chiayi, Taiwan

**Keywords:** atrial fibrillation, atrial flutter, heart failure, stroke

## Abstract

**Aims:**

The clinical outcome and threshold of oral anticoagulation differs between patients with solitary atrial flutter (AFL) and those with AFL developing atrial fibrillation (AF) (AFL-DAF). We therefore investigated previously unevaluated predictors of AF development in patients with AFL, and also the predictive values of risk scores in predicting the occurrence of AF and ischaemic stroke.

**Methods and results:**

Participants were those diagnosed with AFL between 1 January 2001 and 31 December 2013. Patients were classified into solitary AFL and AFL-DAF groups during follow-up. Finally, 4101 patients with solitary AFL and 4101 patients with AFL-DAF were included after 1:1 propensity score matching with CHA_2_DS_2_-VASc scores and their components, AFL diagnosis year and other comorbidities. The group difference in the prevalence of ischaemic stroke/transient ischaemic attack (TIA) and congestive heart failure (CHF) was substantial, that of vascular disease was moderate, and that of diabetes and hypertension was negligible. Therefore, we reweighted the component of heart failure as 2 (the same with stroke/TIA) and vascular disease as 1 in the proposed A_2_C_2_S_2_-VASc score. The proposed A_2_C_2_S_2_-VASc and CHA_2_DS_2_-VAS_C_ scores showed patients with AFL who had higher delta scores and follow-up scores had higher risk of AF development. The delta score outperformed the follow-up score in both scoring systems in predicting ischaemic stroke.

**Conclusion:**

This study showed that new-onset CHF, stroke/TIA and vascular disease were predictors of AF development in patients with AFL. The dynamic score and changes in both CHA_2_DS_2_-VAS_C_ and the proposed A_2_C_2_S_2_-VASc score could predict the development of AF and ischaemic stroke.

**Key questions**

**What is already known about this subject?**

- The clinical outcome and threshold of oral anticoagulation differs between patients with solitary atrial flutter (AFL) and those with AFL developing atrial fibrillation (AF).

**What does this study add?**

- This study showed that new-onset congestive heart failure, stroke/transient ischaemic attack and vascular disease were predictors of AF development in patients with AFL.

**How might this impact on clinical practice?**

- The dynamic score and changes in both CHA_2_DS_2_-VAS_C_ and the proposed A_2_C_2_S_2_-VASc score could predict the development of AF and ischaemic stroke.

## Introduction

Solitary atrial flutter (AFL) and AFL developing into atrial fibrillation (AF) (AFL-DAF) contribute to different levels of mortality, stroke and heart failure (HF).^1–4^ In addition, the threshold of initiation of oral anticoagulation (OAC) to prevent stroke, transient ischaemic attack (TIA) and systemic embolism also differed among these patients.^3 4^ Hence, it would be important to be able to predict those patients with AFL who will develop AF during follow-up. The CHA_2_DS_2_-VASc score (HF, hypertension, age ≥75 (doubled), diabetes, stroke (doubled), vascular disease, age 65–74 and sex category (female)) has been the most commonly used risk score to predict stroke, and its application has been extended to predicting endpoints other than stroke.^5 6^ A previous study reported that the CHA_2_DS_2_-VASc score could also be used to predict the occurrence of AF and ischaemic stroke after cavotricuspid isthmus (CTI) ablation for AFL.^7 8^ Besides, the dynamic changes in the CHA_2_DS_2_-VASc score due to ageing and the new onset of comorbidities should be a concern in predicting the development of AF during follow-up. In addition, the weight of the predicting power of each component of CHA_2_DS_2_-VASc is equal, or may differ with time, so this may be another interesting issue. Therefore, determining the best scoring system to predict the development of AF in patients with AFL among the CHA_2_DS_2_-VASc, the adjusted CHA_2_DS_2_-VASc and even among changes between baseline and the point at which AF is diagnosed is an interesting issue. In this study, we compared these different scores to predict the new development of AF in patients with AFL using a large-scale national cohort.

## Methods

### Data source

This national cohort study used data on patients with AFL retrieved from Taiwan’s National Health Insurance Research Database (NHIRD) from 1 January 2001 to 31 December 2013. The NHIRD is part of Taiwan’s National Health Insurance programme, which is a compulsory single-payer healthcare system covering more than 20 million Taiwanese, and contains healthcare information dating back to 1997. The NHIRD includes data on any outpatient visits, hospitalisation, drug prescriptions, comorbidities and vital status. The identifying data of all participants have been encrypted to protect their privacy, but the participants can be longitudinally followed since the encrypting procedure was consistent. Diseases were identified using the International Classification of Diseases, Ninth Revision, Clinical Modification (ICD-9-CM) codes (online supplemental table 1). Our study population was coded as 427.32, those who were diagnosed as having AFL in consecutive clinics or once at admission. The subpopulation of AF after AFL was also captured (ICD-9-CM: 427.31). The diagnostic codes of both AFL and AF have been validated in previous NHIRD studies.^2 9^

10.1136/openhrt-2020-001478.supp1 Supplementary data

### Study design

**Figure 1:**
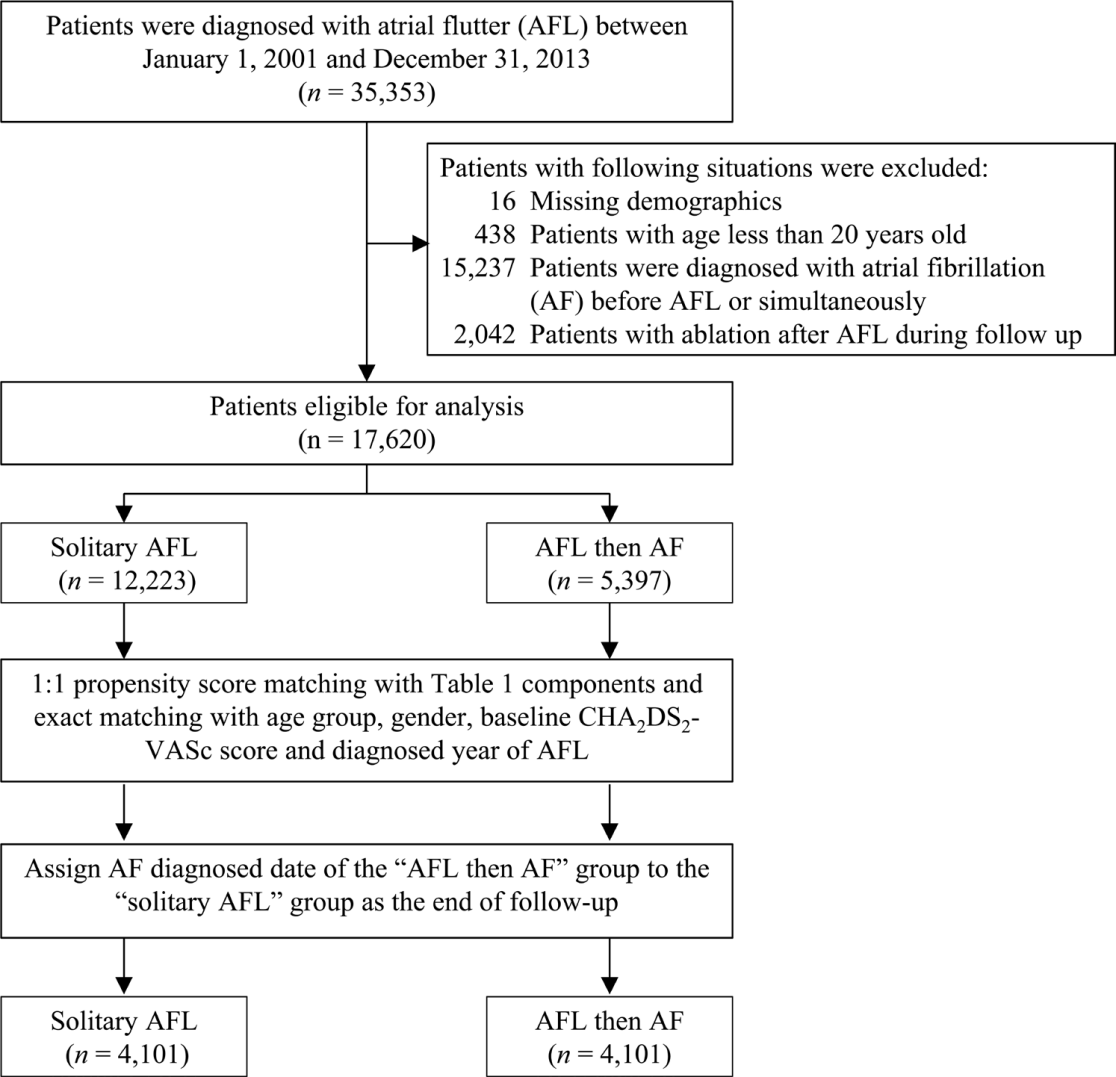
Flow chart of the study design and patient inclusion.

A total of 35 353 patients with AFL between 1 January 2001 and 31 December 2013 were identified for this study. Patients who had missing information or who were <20 years old were excluded. In order to evaluate the post-AFL incidence of AF, patients who were diagnosed with AF prior to the AFL diagnostic date or simultaneously were excluded. Since catheter ablation of AFL may influence the occurrence of AF, patients who received catheter ablation for AFL during follow-up were also excluded. At last, 17 620 patients with AFL were eligible for analysis. The patients with AFL were classified into patients with solitary AFL (n=12 223) and patients with AFL-DAF (n=5397). For patients with AFL that developed AF within the last 3 years, the follow-up duration was from the AFL diagnostic date to the subsequent AF diagnostic date. For a solitary AFL patient, the end date of follow-up was assigned based on the AF diagnostic date of his/her counterpart in the AFL-DAF group. The patients with solitary AFL were restricted to those who survived during follow-up. After 1:1 ratio propensity score matching (PSM), 4101 patients in both groups were included for final analysis (figure 1).

### CHA_2_DS_2_-VAS_C_ score and the proposed A_2_C_2_S_2_-VASc score

The CHA_2_DS_2_-VASc, the components of which include congestive heart failure (CHF), hypertension, age, diabetes, stroke, vascular disease and female, was used to predict the incidence of stroke in non-valvular AF; it was also used to predict the incidence of AF in previous studies.^7 8^ The A_2_C_2_S_2_-VASc is derived from the CHA_2_DS_2_-VASc, and the components are age, CHF, stroke, vascular disease and female sex. These variables were derived from the components of CHA_2_DS_2_-VASc and were reassigned a scoring weight based on the risk ratio of developing AF after AFL (details can be found in the Statistical analyses and Results sections). The follow-up CHA_2_DS_2_-VASc score was calculated based on the patient’s condition at the end of 3 years (for the solitary AFL group) or the time when new AF (for the AFL-DAF group) was noted. The delta score represents the differences between baseline and the end of follow-up. In addition, most of the diagnostic codes for the comorbidities included in the CHA_2_DS_2_-VASc score have been validated with acceptable accuracy in previous NHIRD studies.^10–13^

### Statistical analyses

Each patient in the AFL-DAF group was matched to a counterpart in the solitary AFL group using PSM. The propensity score was the predicted probability to be in the AFL-DAF group given values of covariates using multivariate logistic regression analysis without considering interaction effects. The variables selected to calculate the propensity score were the CHA_2_DS_2_-VASc score and its components, chronic obstructive pulmonary disease and the HATCH score (which is based on HF, hypertension, age ≥75, TIA or stroke, and chronic obstructive pulmonary disease), six other comorbidities and the year of AFL diagnosis. The matching was processed using a greedy nearest neighbour algorithm with a calliper 0.2 times the SD of the logit of the propensity score, with a random matching order and without replacement.

The differences in clinical characteristics and in the scores of interest between groups were evaluated using the absolute value of the standardised difference (STD), where an absolute value of less than 0.1 (−0.1 to 0.1) indicates a negligible difference, an absolute value ranging from 0.1 to 0.2 represents a moderate difference, and an absolute value exceeding 0.2 means a substantial difference.^14^ The follow-up and delta scores of the CHA_2_DS_2_-VASc and the proposed A_2_C_2_S_2_-VASc were used to predict the risk of subsequent AF development. Using the 0 score subgroup as the reference category, the relative risk and corresponding 95% CIs were calculated for each score greater than 0. The area under the curve (AUC), integrated discrimination index (IDI) and net reclassification index (NRI) were used to evaluate the performance of discriminating a subsequent occurrence of ischaemic stroke (defined as the principal diagnosis of hospitalisation) for the matched patients with AFL between the follow-up and delta scores and between the CHA_2_DS_2_-VASc and the proposed A_2_C_2_S_2_-VASc scores. A p value of <0.05 was considered statistically significant. Data analysis was conducted using commercial software (SAS V.9.4).

### Patient and public involvement

Since this study is a retrospective cohort study based on Taiwan national insurance database, in no part or stage of the research were the patients/public involved.

## Results

### Patient inclusion

A total of 17 620 patients were included in the study. Among them, 12 223 had solitary AFL and 5397 developed AF during follow-up (mean and median duration: 0.81 and 0.47 years, respectively). Then, a 1:1 PSM with age group, gender, baseline CHA_2_DS_2_-VASc score, AFL diagnosed year and all components in table 1 was carried out. Moreover, the index date of a patient in the solitary AFL group was assigned based on a counterpart in the AFL-DAF group. Finally, 4101 patients with solitary AFL and 4101 patients with AFL-DAF were eligible for analysis (figure 1).

**Table 1:** Baseline characteristics of study patients before and after propensity score matching

Variables	All patients			Propensity score matched		
	Solitary AFL (n=12 223)	AFL then AF (n=5397)	STD	Solitary AFL (n=4101)	AFL then AF (n=4101)	STD
CHA_2_DS_2_-VASc components						
Female sex	4867 (39.8)	2060 (38.2)	0.03	1570 (38.3)	1570 (38.3)	<0.01
Age (years)	68.7±14.9	71.3±12.5	−0.19	70.1±12.5	70.4±12.5	−0.02
Age group						
<65 (score 0)	4225 (34.6)	1458 (27.0)	0.16	1212 (29.6)	1212 (29.6)	<0.01
65–74 (score 1)	3175 (26.0)	1533 (28.4)	−0.06	1201 (29.3)	1201 (29.3)	<0.01
≥75 (score 2)	4823 (39.5)	2406 (44.6)	−0.10	1688 (41.2)	1688 (41.2)	<0.01
Heart failure	1622 (13.3)	916 (17.0)	−0.10	492 (12.0)	521 (12.7)	−0.02
Hypertension	6987 (57.2)	3231 (59.9)	−0.06	2383 (58.1)	2382 (58.1)	<0.01
Diabetes mellitus	2295 (18.8)	999 (18.5)	0.01	771 (18.8)	677 (16.5)	0.06
Stroke/thromboembolism	1829 (15.0)	848 (15.7)	−0.02	530 (12.9)	547 (13.3)	−0.01
Ischaemic stroke	1611 (13.2)	749 (13.9)	−0.02	475 (11.6)	486 (11.9)	−0.01
Systemic thromboembolism	172 (1.4)	93 (1.7)	−0.03	34 (0.8)	61 (1.5)	−0.06
Venous thromboembolism	133 (1.1)	43 (0.8)	0.03	35 (0.9)	24 (0.6)	0.03
Vascular disease	4529 (37.1)	2273 (42.1)	−0.10	1658 (40.4)	1690 (41.2)	−0.02
Myocardial infarction	647 (5.3)	263 (4.9)	0.02	199 (4.9)	176 (4.3)	0.03
Peripheral arterial disease	316 (2.6)	135 (2.5)	0.01	166 (4.0)	176 (4.3)	−0.01
Ischaemic heart disease	4284 (35.0)	2172 (40.2)	−0.11	1557 (38.0)	1583 (38.6)	−0.01
CHA_2_DS_2_-VASc score	3.0±1.8	3.2±1.8	−0.13	3.1±1.7	3.1±1.7	<0.01
CHA_2_DS_2_-VASc score classification						
0	923 (7.6)	353 (6.5)	0.04	305 (7.4)	305 (7.4)	<0.01
1	1920 (15.7)	582 (10.8)	0.15	506 (12.3)	506 (12.3)	<0.01
2	2267 (18.5)	976 (18.1)	0.01	776 (18.9)	776 (18.9)	<0.01
3	2428 (19.9)	1168 (21.6)	−0.04	916 (22.3)	916 (22.3)	<0.01
4	2178 (17.8)	1016 (18.8)	−0.03	780 (19.0)	780 (19.0)	<0.01
5	1340 (11.0)	693 (12.8)	−0.06	478 (11.7)	478 (11.7)	<0.01
6	688 (5.6)	382 (7.1)	−0.06	218 (5.3)	218 (5.3)	<0.01
7	350 (2.9)	169 (3.1)	−0.02	94 (2.3)	94 (2.3)	<0.01
8	110 (0.9)	44 (0.8)	0.01	23 (0.6)	23 (0.6)	<0.01
9	19 (0.2)	14 (0.3)	−0.02	5 (0.1)	5 (0.1)	<0.01
COPD (included in HATCH score)	2223 (18.2)	1117 (20.7)	−0.06	712 (17.4)	797 (19.4)	−0.05
HATCH score*	1.6±1.3	1.7±1.3	−0.12	1.5±1.3	1.6±1.3	−0.03
HATCH score classification						
0	2764 (22.6)	964 (17.9)	0.12	867 (21.1)	842 (20.5)	0.02
1	3965 (32.4)	1707 (31.6)	0.02	1387 (33.8)	1367 (33.3)	0.01
2	2806 (23.0)	1346 (24.9)	−0.05	1038 (25.3)	1028 (25.1)	0.01
3	1461 (12.0)	771 (14.3)	−0.07	478 (11.7)	505 (12.3)	−0.02
4	843 (6.9)	425 (7.9)	−0.04	234 (5.7)	265 (6.5)	−0.03
5	323 (2.6)	150 (2.8)	−0.01	80 (2.0)	80 (2.0)	<0.01
6	61 (0.5)	34 (0.6)	−0.02	17 (0.4)	14 (0.3)	0.01
Other comorbidities						
Dyslipidaemia	1478 (12.1)	607 (11.2)	0.03	494 (12.0)	439 (10.7)	0.04
Gout	1246 (10.2)	623 (11.5)	−0.04	460 (11.2)	464 (11.3)	<0.01
Renal function status						
Normal	10 449 (85.5)	4672 (86.6)	−0.03	3565 (86.9)	3594 (87.6)	−0.02
Non-dialysis CKD	1380 (11.3)	590 (10.9)	0.01	421 (10.3)	409 (10.0)	0.01
Dialysis	394 (3.2)	135 (2.5)	0.04	115 (2.8)	98 (2.4)	0.03
Immune disease	232 (1.9)	105 (1.9)	<0.01	92 (2.2)	80 (2.0)	0.02
Abnormal liver function	1408 (11.5)	605 (11.2)	0.01	458 (11.2)	462 (11.3)	<0.01
Malignancy	952 (7.8)	378 (7.0)	0.03	260 (6.3)	289 (7.0)	−0.03

Continuous data are presented as mean±SD.

*HATCH score: based on heart failure, hypertension, age ≥75, transient ischemic attack or stroke, and chronic obstructive pulmonary disease

AF, atrial fibrillation; AFL, atrial flutter; CKD, chronic kidney disease; COPD, chronic obstructive pulmonary disease; STD, standardised mean difference.

### Baseline characteristics of patients with solitary AFL and patients with AFL-DAF

The baseline characteristics of the patients with solitary AFL and those with AFL-DAF before and after PSM are shown in table 1. There was no substantial difference between groups after PSM, and all the absolute values of STD were less than 0.1. The distribution of duration from the index date to the end of follow-up between groups before and after PSM can be seen in online supplemental figure 1. The distribution pattern was very similar between groups after PSM.

10.1136/openhrt-2020-001478.supp2 Supplementary data

### CHA_2_DS_2_-VASc components at baseline, during follow-up, and delta values between patients with solitary AFL and patients with AFL-DAF

At baseline, there were no differences between groups. During the 3-year follow-up, the patients with AFL-DAF had more HF, stroke/TIA, and vascular disease, and higher CHA_2_DS_2_-VASc scores (STD values <−0.1). Since the group differences in incremental prevalence of HF and stroke/TIA was substantial (STD values <−0.2), that of vascular disease was moderate (STD values ranged from −0.2 to −0.1), and that of diabetes and hypertension was negligible (STD values ranged from −0.1 to 0.1), we reweighted the component of HF as 2 (the same with stroke/TIA), vascular disease as 1, and diabetes/hypertension as none in the proposed A_2_C_2_S_2_-VASc score (table 2).

**Table 2:** Differences in CHA_2_DS_2_-VASc components at baseline and during follow-up, and the delta values of patients with solitary AFL and AFL developing into AF

CHA_2_DS_2_-VASc components	At baseline			During follow-up			Delta (follow-up—baseline)		
	Solitary AFL (n=4101)	AFL then AF (n=4101)	STD	Solitary AFL (n=4101)	AFL then AF (n=4101)	STD	Solitary AFL (n=4101)	AFL then AF (n=4101)	STD
Female	1570 (38.3)	1570 (38.3)	<0.01	－	－	－	－	－	－
Age group							－	－	－
20–64 (score 0)	1212 (29.6)	1212 (29.6)	<0.01	1161 (28.3)	1134 (27.7)	0.02	－	－	－
65–74 (score 1)	1201 (29.3)	1201 (29.3)	<0.01	1159 (28.3)	1168 (28.5)	−0.02	－	－	－
≥75 (score 2)	1688 (41.2)	1688 (41.2)	<0.01	1781 (43.4)	1799 (43.9)	−0.02	－	－	－
Delta of age				－	－	－	0.04±0.18	0.05±0.21	−0.06
Heart failure	492 (12.0)	521 (12.7)	−0.02	874 (21.3)	1428 (34.8)	−0.30	382 (9.3)	907 (22.1)	−0.36
Hypertension	2383 (58.1)	2382 (58.1)	<0.01	2762 (67.3)	2824 (68.9)	−0.03	379 (9.2)	442 (10.8)	−0.05
Diabetes mellitus	771 (18.8)	677 (16.5)	0.06	876 (21.4)	775 (18.9)	0.06	105 (2.6)	98 (2.4)	0.01
Stroke/SE	530 (12.9)	547 (13.3)	−0.01	732 (17.8)	1068 (26.0)	−0.20	202 (4.9)	521 (12.7)	−0.28
Vascular disease	1658 (40.4)	1690 (41.2)	−0.02	2137 (52.1)	2406 (58.7)	−0.13	479 (11.7)	716 (17.5)	−0.16
CHA_2_DS_2_-VASc score	3.1±1.7	3.1±1.7	<0.01	3.5±1.8	3.9±1.8	−0.20	0.46±0.78	0.83±1.01	−0.40
Proposed A_2_C_2_S_2_-VASc score	2.4±1.6	2.4±1.6	−0.02	2.8±1.8	3.3±1.9	−0.28	0.44±0.84	0.92±1.17	−0.47

AF, atrial fibrillation; AFL, atrial flutter; SE, thromboembolism; STD, standardised mean difference.

**Figure 2:**
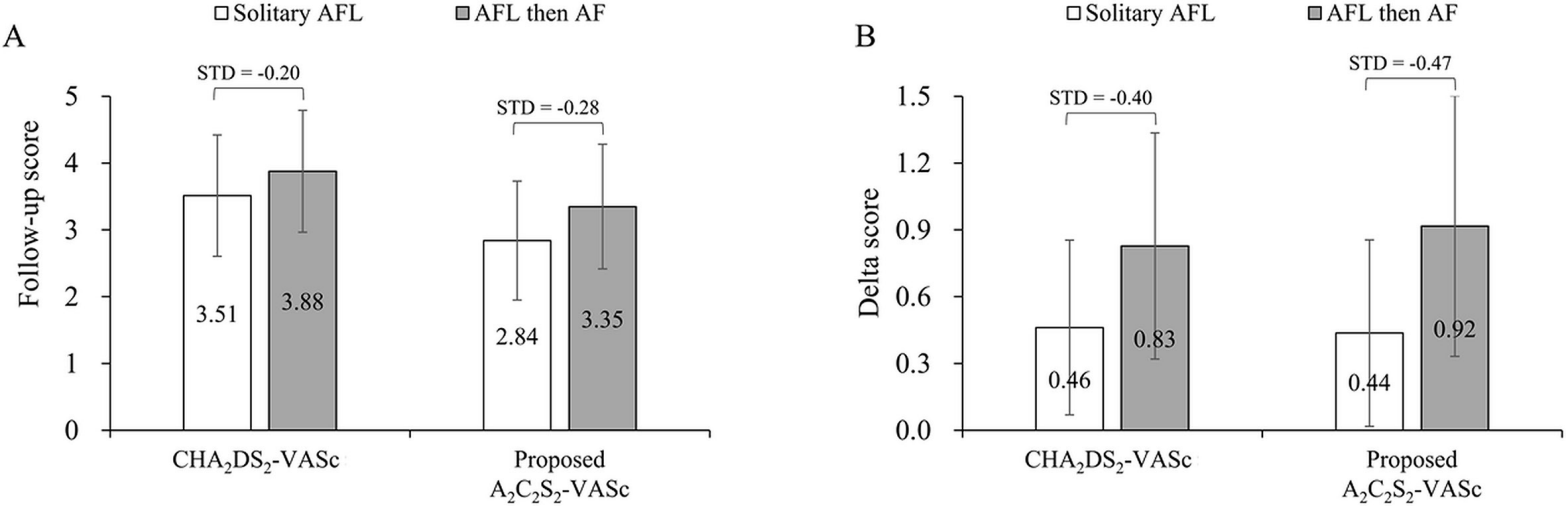
The mean CHA_2_DS_2_-VASc and proposed A_2_C_2_S_2_-VASc scores of patients with solitary AFL and AFL then AF. (A) Follow-up score of both scoring systems. (B) Delta score of both scoring systems. The error bar represents the SD. AF, atrial fibrillation; AFL, atrial flutter; STD, standardised difference.

Therefore, the A_2_C_2_S_2_-VASc score was (age ≥75 (doubled), CHF (doubled), stroke (doubled), vascular disease, age 65–74 and sex category (female)). The follow-up A_2_C_2_S_2_-VASc score and delta A_2_C_2_S_2_-VASc score were substantially higher in patients with AFL-DAF than in those with solitary AFL (STD values <−0.2). Furthermore, the CHA_2_DS_2_-VASc and A_2_C_2_S_2_-VASc scores during follow-up were significantly higher in the AFL-DAF group than in the solitary AFL group (figure 2A). And, the delta score of CHA_2_DS_2_-VASc and the A_2_C_2_S_2_-VASc score were significantly higher in the AFL-DAF group than in the solitary AFL group (figure 2B).

### Distribution of components for the CHA_2_DS_2_-VASc score and the A_2_C_2_S_2_-VASc score between patients with solitary AFL and patients with AFL-DAF

**Figure 3:**
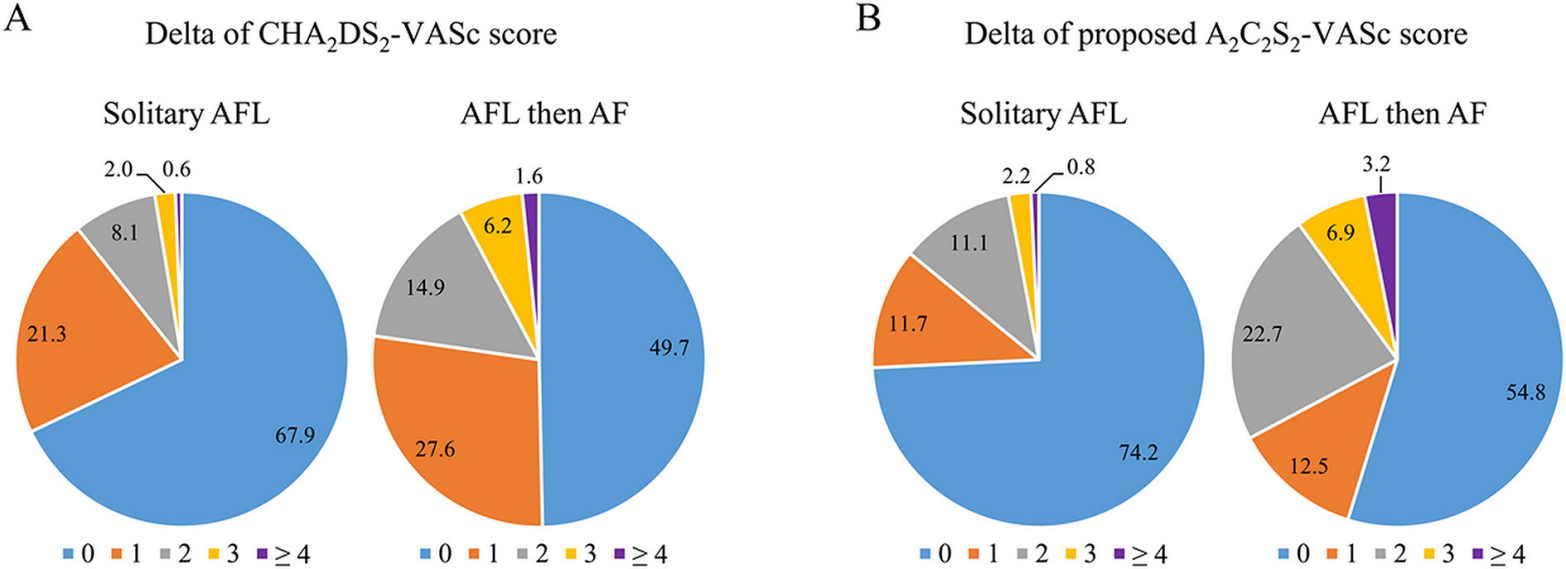
Distribution of components for the delta CHA_2_DS_2_-VASc score (A) and the delta A_2_C_2_S_2_-VASc score (B) between solitary AFL and AFL developing into patients with AF. AF, atrial fibrillation; AFL, atrial flutter.

The distribution of delta CHA_2_DS_2_-VASc scores in the solitary AFL group and the AFL-DAF group showed the percentage of delta scores ≥1 was higher in the AFL-DAF group than in the solitary AFL group (50.3% vs 32.1%) (figure 3A). The distribution of delta A_2_C_2_S_2_-VASc scores in the solitary AFL group and the AFL-DAF group also showed that the percentage of delta scores ≥1 was higher in the AFL-DAF group than in the solitary AFL group (45.2% vs 25.8%) (figure 3B).

### Predicting development of AF using the CHA_2_DS_2_-VASc score and the A_2_C_2_S_2_-VASc score in patients with AFL

**Figure 4:**
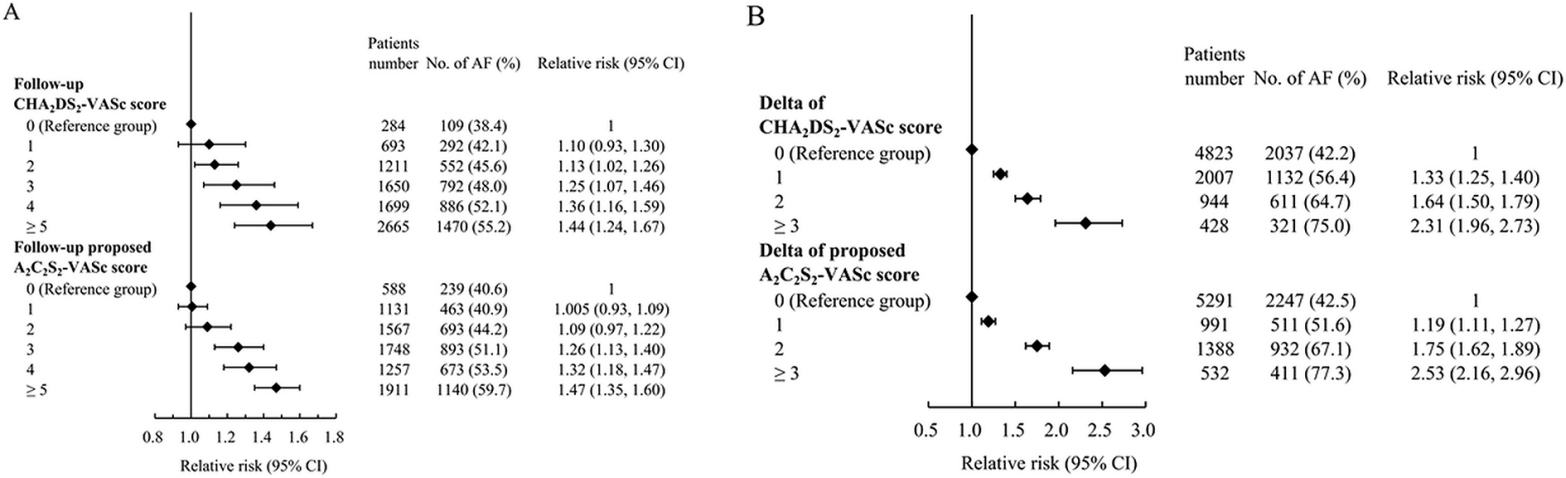
Relative risk of predicting atrial flutter developing AF using different follow-up scores (A) and delta scores (B) of the CHA_2_DS_2_-VASc scoring system and the proposed A_2_C_2_S_2_-VASc scoring system. AF, atrial fibrillation.

The event rate of AF development at different levels of follow-up CHA_2_DS_2_-VASc scores and follow-up A_2_C_2_S_2_-VASc scores can be seen in figure 4A. The risks of AF development in patients with follow-up CHA_2_DS_2_-VASc scores ≥2 and in patients with follow-up scores ≥3 in the proposed A_2_C_2_S_2_-VASc score were significantly higher than in patients with follow-up scores of 0. The effect size (relative risks) at each level of score ≥1 was similar between these two scores. Figure 4B shows the event rate of AF development at different levels of delta CHA_2_DS_2_-VASc scores and delta A_2_C_2_S_2_-VASc scores. The relative risk of AF development in patients with scores ≥1 in both the delta CHA_2_DS_2_-VASc and delta A_2_C_2_S_2_-VASc scoring systems was significantly higher than in patients with a delta score of 0. The effect size (relative risks) at the level of a score ≥2 was slightly higher in the delta A_2_C_2_S_2_-VASc than in the delta CHA_2_DS_2_-VASc scoring systems.

### Performance of the CHA_2_DS_2_-VASc score and the A_2_C_2_S_2_-VASc score in discriminating subsequent occurrence of ischaemic stroke

Finally, we compared the delta score to the follow-up, and the proposed A_2_C_2_S_2_-VASc score to the CHA_2_DS_2_-VASc score in discriminating the subsequent occurrence of ischaemic stroke in the 8202 matched patients with AFL. The AUC increment, the IDI and NRI were significantly greater in the delta score than in the follow-up score for both the CHA_2_DS_2_-VASc and A_2_C_2_S_2_-VASc scores. The results indicated that the AUC increment, the IDI and NRI were significantly greater in the A_2_C_2_S_2_-VASc score than in the CHA_2_DS_2_-VASc score using both the follow-up and the delta scores (table 3).

**Table 3:** Comparison of delta score to the follow-up score as well as the proposed A_2_C_2_S_2_-VASc score to CHA_2_DS_2_-VASc score for discriminating a subsequent occurrence of ischaemic stroke in the 8202 matched patients with atrial flutter

Score	AUC, % (95% CI)	Delta AUC, % (95% CI)	IDI, % (95% CI)	Category-free NRI, % (95% CI)
CHA_2_DS_2_-VASc				
Follow-up	55.6 (54.4 to 56.8)	Reference	Reference	Reference
Delta	60.2 (59.1 to 61.3)	4.60 (3.23 to 5.96)	2.97 (2.56 to 3.37)	25.9 (21.6 to 30.1)
Proposed A_2_C_2_S_2_-VASc				
Follow-up	57.9 (56.7 to 59.2)	Reference	Reference	Reference
Delta	61.0 (60.0 to 62.0)	3.05 (1.84 to 4.27)	3.40 (2.96 to 3.84)	22.2 (17.9 to 26.5)
Follow-up				
CHA_2_DS_2_-VASc	55.6 (54.4 to 56.8)	Reference	Reference	Reference
Proposed A_2_C_2_S_2_-VASc	57.9 (56.7 to 59.2)	2.34 (1.80 to 2.87)	0.93 (0.79 to 1.06)	25.6 (21.3 to 29.8)
Delta				
CHA_2_DS_2_-VASc	60.2 (59.1 to 61.3)	Reference	Reference	Reference
Proposed A_2_C_2_S_2_-VASc	61.0 (60.0 to 62.0)	0.79 (0.31 to 1.27)	1.36 (1.13 to 1.59)	35.4 (31.9 to 38.9)

All the results were significant at a p<0.001 level.

AUC, area under the curve; IDI, integrated discrimination index; NRI, net reclassification index.

## Discussion

There are several important findings in this study. First, the percentages of new-onset CHF, stroke/TIA and peripheral artery disease were different in patients with solitary AFL and those with AFL-DAF. Second, the incidence of AF development was higher in patients with AFL with follow-up CHA_2_DS_2_-VASc scores ≥2 and A_2_C_2_S_2_-VASc scores ≥3. Third, the incidence of AF development was higher in patients with AFL with delta scores ≥1 in both scoring systems compared with those with a delta score=0. Finally, the delta score system was better than the follow-up score in predicting ischaemic stroke. In addition, the follow-up score of the reweighted A_2_C_2_S_2_-VASc score was better than the follow-up score of the original CHA_2_DS_2_-VASc score in predicting ischaemic stroke.

### Risk factors for patients with AFL developing AF during follow-up

According to the Framingham heart study follow-up data and also the meta-analysis, several risk factors, including male sex, ageing, hypertension, CHF, coronary artery disease, valvular heart disease and diabetes mellitus were associated with AF development.^15 16^ Among these risk factors, CHF, male sex and coronary artery disease were the three strongest risk factors for predicting the development of AF.^16^ In the present study, the incidence of new-onset CHF, stroke and vascular disease was higher in patients with AFL who developed AF during follow-up than in those who did not have AF development. Our study also showed that the relative risk of new-onset CHF in patients developing AF was the same as that of new-onset stroke in patients with AFL, and was higher than that of developing new-onset vascular disease. Those patients with AFL at a low level of CHA_2_DS_2_-VASc who suffered from these diseases during follow-up should accept more aggressive monitoring and follow-up in the diagnosis of AF, because the strategy of OAC use and clinical outcome might be different.

### Scoring systems for predicting development of AF in patients with AFL

Some scoring systems, including the CHA_2_DS_2_-VASc score, have been used to predict new-onset AF in typical patients with AFL receiving catheter ablation for CTI, and also to predict long-term mortality in typical AFL.^7 8 17 18^ To the best of our knowledge, there is no scoring system to predict AF and stroke development in patients with AFL. Our study, using a large nationwide database, reveals the independent risk factors for AF occurrence during follow-up in patients with AFL. Our study also showed that both the follow-up score and the delta score of both scoring systems can predict AF development in patients with AFL. In addition, the delta scoring system is stronger than the follow-up scoring system in predicting AF development. A delta score ≥3 was more powerful than a follow-up score ≥5, and indicated an at least 1.6-fold increased risk of developing AF. Therefore, closely monitoring and following up patients with AFL who have a new-onset of comorbidities to detect the occurrence of AF is as important as the management of the comorbidity itself, particularly a new attack of HF and stroke.

### Comparing the ability of scoring systems to predict the development of ischaemic stroke in patients with AFL

According to our study, the A_2_C_2_S_2_-VASc follow-up score was better than the CHA_2_DS_2_-VASc score in predicting ischaemic stroke development in patients with AFL, and there was no difference in the delta score of both scoring systems in predicting ischaemic stroke. Even more, the delta score was a stronger predictor of ischaemic stroke than the follow-up score in both scoring systems, and the delta score had improved NRI compared with the follow-up score. A previous study showed that the CHA_2_DS_2_-VASc score was not static in the AF cohort, and that stroke risk in patients with AF is a dynamic process due to increasing age and incident comorbidities.^19^ Our study emphasised that the risk of ischaemic stroke in patients with AFL may continuously increase, and that regularly reassessing the scoring systems and the strategy of stroke prevention during follow-up in these patients with AFL are also important.

### Study limitations

There are some limitations in this retrospective insurance database study. First, the subtype of AFL was not recorded in the NHIRD database. So, we could not clearly classify the types of AFL (neither CTI vs non-CTI flutter, nor left-side AFL vs right-side AFL), which may also have an impact on the occurrence of AF during follow-up. However, we could not confirm the actual type and mechanism of AFL in those patients who did not receive ablation, and the success or failure of ablation could also influence the occurrence of AF thereafter. So, this study evaluated the potential risks of AF development in all patients with AFL, including those patients who did not receive ablation. Second, although our study, using the CHA_2_DS_2_-VASc score and also the reweighted A_2_C_2_S_2_-VASc score, showed patients with a higher score had a higher risk of AF development, we did not evaluate the anticoagulation status or other medications used for disease control and risk modification before and after AF development. We could not evaluate the quality of care provided for other new-onset comorbidities before the diagnosis of AF or the use and adherence to anticoagulation medications after the diagnosis of AF, which may influence not only the risk of AF development, but also the risk of ischaemic stroke.

## Conclusions

This national cohort study showed that new-onset CHF, stroke/TIA and vascular disease were predictors of AF development in patients with AFL. Patients with AFL and delta scores ≥1 in both the CHA_2_DS_2_-VASc and A_2_C_2_S_2_-VASc scoring systems had a higher risk of AF development during follow-up. Meanwhile, patients with AFL and a follow-up score ≥2 in the CHA2DS2-VASc and ≥3 in the A_2_C_2_S_2_-VASc scoring systems also had a higher risk of AF development during follow-up. The delta score was better than the follow-up score in both scoring systems in predicting ischaemic stroke during follow-up. Patients with AFL at a low level of CHA_2_DS_2_-VASc should be closely followed up when a newly developed comorbidity of a component of the CHA_2_DS_2_-VASc is noted thereafter.
