# Supplementary material for: Proposed A2C2S2-VASc score for predicting atrial fibrillation development in patients with atrial flutter
Source: Open Heart. 2021 Jan 29;8(1):e001478. doi: 10.1136/openhrt-2020-001478 (PMC7849887; doi:10.1136/openhrt-2020-001478)
Supplement: Supplementary data [file openhrt-2020-001478supp001.pdf]

Supplemental Table 1. Diagnoses used to define the cohorts, comorbidities, and outcomes

Disease	Diagnostic criteria	ICD-9 CM disease code
Study Cohorts		
Atrial flutter	Defined from diagnosis	427.32
Excluded Diseases		
Atrial fibrillation	Defined from diagnosis	427.31
CHA_2DS_2-VASc and HATCH components		
Heart failure	Defined from principle diagnosis in hospital	428
Hypertension	Defined from diagnosis plus treatment	401, 402, 403, 404, 405 and any anti-hypertension drugs
Diabetes mellitus	Defined from diagnosis plus treatment	250 and any oral hypoglycemic drugs and insulin
Ischemic stroke	Defined from principle diagnosis in hospital	433 ~ 437
Systemic thromboembolism	Defined from diagnosis in hospital	415.1x, 444.22, 444.81, 444.21, 362.30, 362.34, 593.81, 444.89, 557.0, 557.9, 557.1, 444.9x
Venous thromboembolism	Defined from diagnosis	415.1x
Myocardial infarction	Defined from principle diagnosis in hospital	410 ~ 412
Peripheral arterial disease	Defined from diagnosis	093.0, 437.3, 440.x, 441.x, 443.x, 444.0x, 444.22, 444.8x, 447.1, 447.8x, 447.9x, 557.1, 557.9, V43.4
Ischemic heart disease	Defined from diagnosis	410 ~ 414
Chronic obstructive pulmonary disease	Defined from diagnosis	491, 492, 496
Other comorbidities		
Dyslipidemia	Defined from diagnosis plus treatment	272 and any lipid-lowering agents

Gout	Defined from diagnosis	274
Chronic kidney disease	Defined from diagnosis	580 ~ 589, 403 ~ 404<br>016.0, 095.4, 236.9 250.4, 274.1, 442.1, 447.3, 440.1, 572.4, 642.1, 646.2 753.1 283.11, 403.01,and 404.02, 446.21
Dialysis	Defined from diagnosis in catastrophic registry data	585
Immune disease	Defined from diagnosis	710.0, 710.1, 714.0, 710.4, 710.3, 446.0, 446.2, 446.4, 446.5, 443.1, 446.7, 136.1, 694.4, 710.2, 555, 556.xx
Abnormal liver function	Defined from diagnosis	070 , 456.0 ~ 456.2, 570, 571, 572.2–572.8, 573, V42.7
Malignancy	Defined from diagnosis in catastrophic registry data	140 ~ 208

ICD-9-CM, International Classification of Diseases, Ninth Revision, Clinical Modification.
