# Supplementary figures and images for: Proposed A2C2S2-VASc score for predicting atrial fibrillation development in patients with atrial flutter
Source: Open Heart. 2021 Jan 29;8(1):e001478. doi: 10.1136/openhrt-2020-001478 (PMC7849887; doi:10.1136/openhrt-2020-001478)

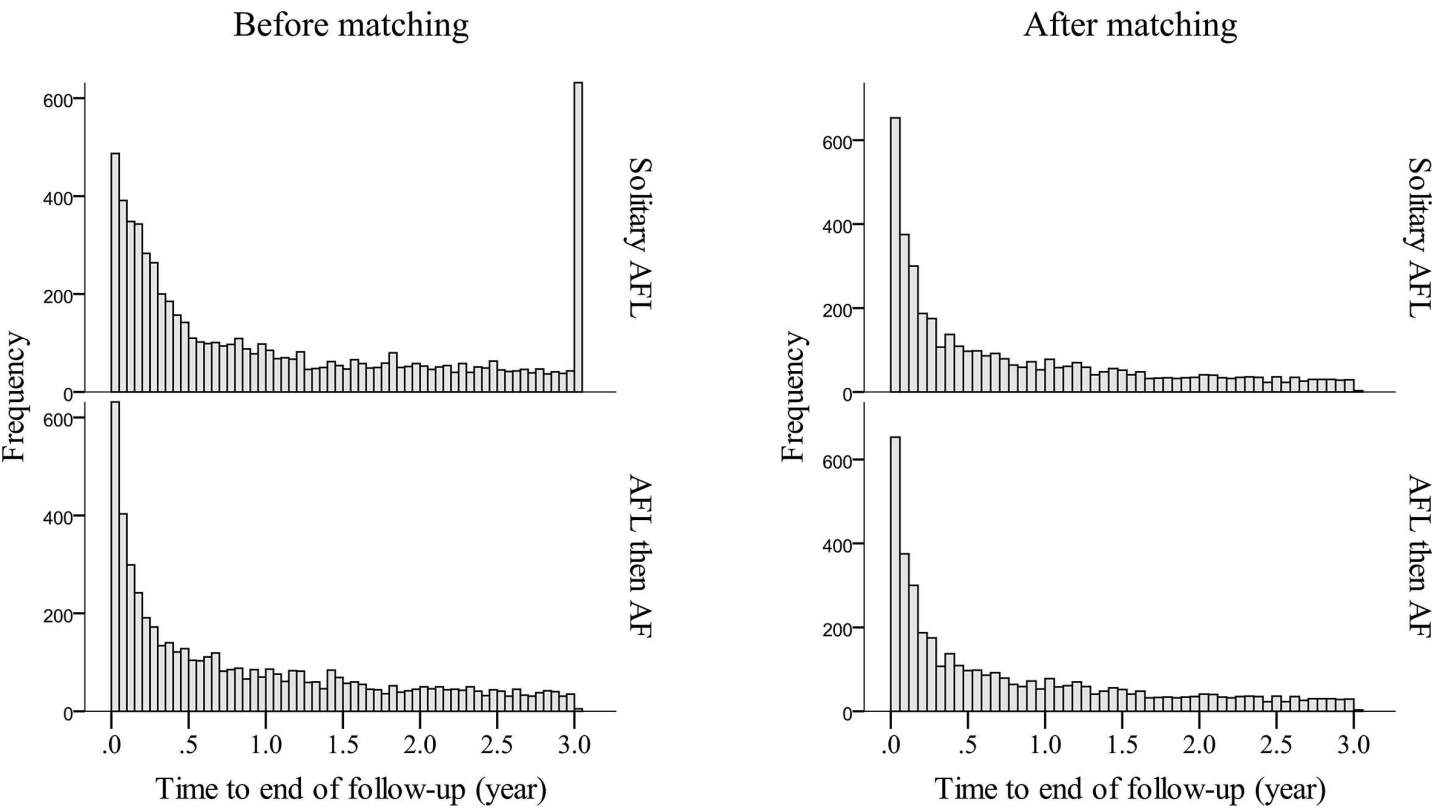

Supplement: Supplementary data [file openhrt-2020-001478supp002.pdf]
